# Integrated Transcriptomic and Metabolomic Analysis Reveals the Regulatory Mechanisms of *Macrobrachium rosenbergii* in Response to Acute High-Salinity Stress

**DOI:** 10.3390/biology15151234

**Published:** 2026-07-24

**Authors:** Yan Xia, Zhonghong Li, Xudong Shen, Mingkang Chen, Yanfeng Zhao, Chuang Gao, Chuanqi Ren, Jianchun Zhang, Shuang Li, Bo Ma

**Affiliations:** 1 Yingkou Enhancement and Experiment Station, Chinese Academy of Fishery Sciences, Yingkou 115004, China; xiayan1207@126.com (Y.X.); lizhonghong12345@126.com (Z.L.); shenxudong_good@163.com (X.S.); chenmingkang1202@126.com (M.C.); 17866542597@163.com (Y.Z.); gaochuang0919@163.com (C.G.); ren24233335@163.com (C.R.); 2 Section of Aquatic Food, Weihai Aquatic School, Rongcheng 264303, China; zhangjianchun227@163.com; 3 Heilongjiang River Fisheries Research Institute, Chinese Academy of Fishery Sciences, Harbin 150070, China

**Keywords:** *Macrobrachium rosenbergii*, transcriptomics and metabolomics, salinity stress, immune response, metabolic regulation

## Abstract

Salinity fluctuations pose a severe threat to the survival and physiological homeostasis of freshwater crustaceans. Here, an integrated transcriptomic and metabolomic approach was utilised to characterise the dynamic regulatory responses in the hepatopancreas of adult *Macrobrachium rosenbergii* subjected to acute high-salinity stress (20‰). The number of differentially expressed genes (DEGs) and metabolites (DMs) increased from 24 to 48 h, signalling a transition from an initial acute stress reaction to systemic homeostatic remodelling. At the molecular level, *M. rosenbergii* activated autophagy (*Atg8a*) and p53 (*TP53INP*) signalling, alongside the reprogramming of lipid and nucleotide metabolism. Pathway analysis identified “alanine, aspartate, and glutamate metabolism” as a central regulatory node. Notably, the downregulation of the glutamate dehydrogenase gene (*Gdh*) alongside the accumulation of L-glutamate suggests a potential transcriptional-metabolic decoupling phenomenon, wherein the organism appears to prioritise the conservation of critical osmoregulatory effectors over their oxidative degradation. These findings detail the systemic adaptive responses of *M. rosenbergii* to salinity fluctuations, providing candidate targets for the selective breeding of stress-resilient crustacean strains.

## Simple Summary

*Macrobrachium rosenbergii* is an economically important species in freshwater aquaculture. However, climate change and rising sea levels are causing sudden increases in salt levels in farming areas, threatening their survival and regional economies. This study aimed to understand the internal survival strategies of adult prawns when exposed to sudden high salt conditions over a two-day period. By examining the active genes and metabolites in the prawns, researchers discovered that the animals shift from a quick stress response to a systemic physical adjustment. The results showed that to survive the salty environment, the prawns activate internal cell cleaning and repair systems while changing how they use fats and energy. Most importantly, they use a unique strategy to reduce the breakdown of specific amino acids, retaining these molecules to balance the intense outside salt pressure. In conclusion, these prawns survive extreme salt stress by combining cellular protection with a metabolite saving strategy. This research not only decodes the survival mechanisms of these prawns but also provides a basis for breeding salt-resistant varieties, helping the aquaculture industry better address the practical challenges posed by climate change.

## 1. Introduction

*Macrobrachium rosenbergii* is an economically important freshwater prawn in tropical and subtropical regions, recognised for its rapid growth, short culture cycle, and nutritional profile [1,2]. However, climate-driven phenomena, particularly sea-level rise and seawater intrusion, are increasingly exposing freshwater farming regions to salinity fluctuations, thereby threatening regional aquaculture economies [3]. As a critical environmental determinant, abrupt salinity changes disrupt osmotic balance and impair growth, development, and reproduction [4,5]. In the salinity-sensitive freshwater prawn *M. rosenbergii*, high-salinity stress inhibits reproductive performance [6], diminishes larval yield and survival [7], and triggers severe physiological stress in vital tissues such as the hepatopancreas [8].

To cope with salinity fluctuations, aquatic animals have evolved complex molecular regulatory networks to maintain homeostasis [9,10]. Research highlights that crustaceans mitigate osmotic stress by modulating heat shock protein (HSP90) expression [11], chitinase activity [12], and amino acid metabolism (e.g., D-alanine and glutamate) [13]. To date, investigations into *M. rosenbergii* have largely been confined to transcriptional alterations [14] or single-omics profiling under alternative stressors, such as ammonia exposure [15]. However, single-omics strategies possess inherent limitations. Because of complex post-transcriptional and post-translational modifications, transcriptomic data often fail to directly correlate with the functional levels of proteins and downstream metabolites. Conversely, metabolomic profiles are sensitive to individual variability and environmental noise, which can compromise data reproducibility. Consequently, relying exclusively on single-omics data is insufficient to fully resolve the systemic responses of these organisms [16,17,18].

To overcome these limitations of single-omics strategies, correlating gene expression with metabolic profiles enables a comprehensive resolution of biological processes, effectively bridging the gap between transcriptional regulation and functional phenotypes [19,20,21]. This multi-omics approach has been instrumental in revealing salinity adaptation mechanisms in various crustaceans, including *Fenneropenaeus chinensis* [22] and *Macrobrachium nipponense* [23]. While previous research on *M. rosenbergii* under salinity stress has primarily targeted specific gene expression patterns or early developmental stages [8,10], the temporal, system-level dynamic responses of adult prawns to acute high-salinity exposure remain poorly defined.

To address this gap, the present study utilised integrated transcriptomics and metabolomics to profile the differentially expressed genes (DEGs), differential metabolites (DMs), and their corresponding regulatory networks in adult *M. rosenbergii* during a time-course of acute high-salinity stress (20‰). By detailing the dynamic molecular defence strategies of freshwater crustaceans against abrupt salinity surges, this research provides essential theoretical insights and candidate biomarkers for the precision breeding and evaluation of stress-resilient strains.

## 2. Materials and Methods

### 2.1. Experimental Animals and Rearing Conditions

Transparent glass tanks (0.8 m × 0.5 m × 0.6 m) were pre-disinfected with 20 mg/L potassium permanganate (Aladdin Biochemical Technology Co., Ltd., Shanghai, China), and the working water volume in each tank was maintained at 200 L (0.5 m depth). *M. rosenbergii* in the intermolt stage (average body weight: 34 ± 2.7 g) were sourced directly from the outdoor ponds of the Yingkou Experimental Station of Propagation, Chinese Academy of Fishery Sciences (Yingkou, China). To eliminate the potential confounding effects of reproductive status and molting, all experimental prawns used in this study were exclusively healthy males and were physically confirmed to be in the intermolt stage (Stage C) prior to the experiment. The prawns were then randomly allocated into the prepared tanks at a stocking density of 250 ind./m^2^.

The prawns underwent an initial 7-day acclimatisation period to ensure a smooth transition from the outdoor pond environment to the controlled indoor laboratory conditions. To minimize handling stress and prevent cannibalism, artificial refuges were provided in all tanks throughout both the acclimatisation and the subsequent experimental periods. During this acclimatisation period, the prawns were fed a commercial formulated diet three times daily, with a total daily ration equivalent to 4% of their body weight. Water quality was maintained by daily siphoning of feces and uneaten feed, followed by a 25% water exchange. Throughout both the acclimatisation and experimental periods, water quality parameters were rigorously maintained at 26 ± 1 °C, dissolved oxygen (DO) at 6 ± 1 mg/L, and pH at 7.0 ± 1.0. Additionally, total ammonia nitrogen (TAN) at 0.08 ± 0.02 mg/L, nitrite at 0.02 ± 0.01 mg/L, and alkalinity at 162 ± 2 mg/L. Prawn survival and general behavior were recorded daily, and any dead individuals were promptly removed.

### 2.2. Experimental Design and Acute Salinity Stress Challenge

Following the one-week acclimation, 216 healthy prawns were fasted for 24 h to evacuate their digestive tracts. The saline water for the challenge was artificially prepared by dissolving commercial artificial sea salt (Yuehai, Zhanjiang, China) into dechlorinated tap water, followed by vigorous aeration for 24 h to ensure complete dissolution. The target salinity was set at 20‰ and calibrated using a portable refractometer (ATAGO, Tokyo, Japan). The prawns were then randomly allocated into two salinity treatments: a freshwater control (0‰) and an acute high-salinity stress group (20‰). To avoid immediate osmotic shock, the salinity for the stress group was not adjusted abruptly; rather, it was gradually increased at a rate of 2‰ per hour until the target salinity of 20‰ was reached. Each treatment was performed in triplicate, with 36 prawns per replicate tank. The stress challenge was maintained for 48 h, with random sampling conducted at 24 and 48 h after reaching the target salinity. Accordingly, the experimental groups were designated as N (freshwater parallel control sampled at 48 h to eliminate temporal confounding effects), P24 (20‰ stress for 24 h), and P48 (20‰ stress for 48 h).

### 2.3. Transcriptomic Analysis

At each sampling time point (24 h and 48 h), one prawn was randomly selected from each of the three replicate tanks per treatment (*n* = 3 biological replicates per group). Following rapid chilling (cold anesthesia) in an ice slurry, hepatopancreas tissues were collected without pooling and subjected to transcriptome sequencing by Biomarker Technologies Corporation (Beijing, China). Total RNA was isolated using the TRIzol reagent according to the manufacturer’s protocol. Sequencing libraries were subsequently constructed and sequenced on an Illumina NovaSeq platform (Illumina, San Diego, CA, USA) to generate 150 bp paired-end reads. Raw reads were filtered to remove adapter sequences, poly-N tracts, and low-quality sequences, yielding high-quality clean reads. These clean reads were then aligned to the *M. rosenbergii* reference genome using HISAT2 [24]. Transcript assembly was performed via StringTie [25], and gene expression levels were quantified as Fragments Per Kilobase of transcript per Million mapped reads (FPKM) [26]. DEGs were identified using the DESeq2 R package (version 1.30.1) [27], applying significance thresholds of |log_2_Fold Change| ≥ 1.5 and *p* < 0.05. The False Discovery Rate (FDR) was derived by adjusting the raw *p*-values, providing a more robust measure of statistical significance. Finally, Kyoto Encyclopedia of Genes and Genomes (KEGG) pathway enrichment analysis and correlation network construction were performed using the KOBAS database [28] and the clusterProfiler R package.

### 2.4. Metabolomic Analysis

At each sampling time point, two prawns were randomly selected from each of the three replicate tanks per treatment (*n* = 6 biological replicates per group). Similarly, following rapid chilling (cold anesthesia) in an ice slurry, hepatopancreas tissues were collected without pooling and subjected to metabolomic profiling by Biomarker Technologies Corporation (Beijing, China). Briefly, samples were homogenized in extraction solvent, followed by sonication and centrifugation. The resulting supernatants were vacuum-dried and reconstituted prior to analysis. Chromatography and mass spectrometry were performed using a Waters Acquity I-Class PLUS ultra-performance liquid chromatography (UPLC) system coupled with a Xevo G2-XS quadrupole time-of-flight (QTOF) mass spectrometer. Raw data, acquired via MassLynx V4.2 software, were processed for peak alignment and feature extraction using Progenesis QI (version 2.0). Metabolite annotation was performed by cross-referencing against the METLIN, HMDB, and KEGG databases, alongside a custom reference library, achieving Metabolomics Standards Initiative (MSI) Level 2 confidence (putatively annotated compounds) [29]. Following quality control (QC) validation, orthogonal partial least squares-discriminant analysis (OPLS-DA) was conducted [30]. The robustness and reliability of the OPLS-DA models were evaluated using the quality parameters R^2^Y (explanatory rate) and Q^2^ (predictive ability), and further validated by 200-iteration permutation tests to ensure the absence of overfitting. DMs were defined based on a variable importance in projection (VIP) score ≥ 1 and *p* < 0.05. Finally, KEGG pathway enrichment and correlation network analyses of the identified DMs were executed.

### 2.5. Integrated Transcriptomic and Metabolomic Analysis

To characterise the system-level responses to salinity stress, DEGs and DMs were concurrently mapped to the KEGG database to identify significantly enriched shared metabolic pathways. Enrichment results were visualized via bar charts, where transcriptomic and metabolomic data were distinguished by color (yellow and blue, respectively). Subsequently, key DEGs and DMs (*p* < 0.05) mapped within these overlapping pathways were isolated to reconstruct integrated metabolic networks using the Pathview R package [31].

### 2.6. Quantitative Real-Time PCR Validation

To validate the RNA-Seq data, seven DEGs were randomly selected for RT-qPCR analysis. Total RNA was reverse-transcribed into cDNA using the TRUEscript First Strand cDNA Synthesis Kit (Aidlab, Beijing, China). Reactions were performed on a qTOWER 2.2 system (Analytik Jena, Germany) using 2× SYBR^®^ Green Supermix (DBI Bioscience, Ludwigshafen, Germany). The thermocycling protocol consisted of an initial denaturation at 95 °C for 3 min, followed by 39 cycles of 95 °C for 10 s and 60 °C for 30 s, concluding with a melting curve analysis (60–95 °C) to verify product specificity. Expression levels were normalized to the 18S rRNA internal control, with all samples run in technical triplicates. Relative expression was calculated via qPCRsoft 3.2 software. Primer sequences are provided in Supplementary Table S1. Statistical analysis of the relative expression levels between groups was performed using Student’s *t*-test, with *p* < 0.05 considered statistically significant.

## 3. Results

### 3.1. Dynamic Transcriptomic Profiling and Identification of DEGs Under High-Salinity Stress

**Figure 1:**
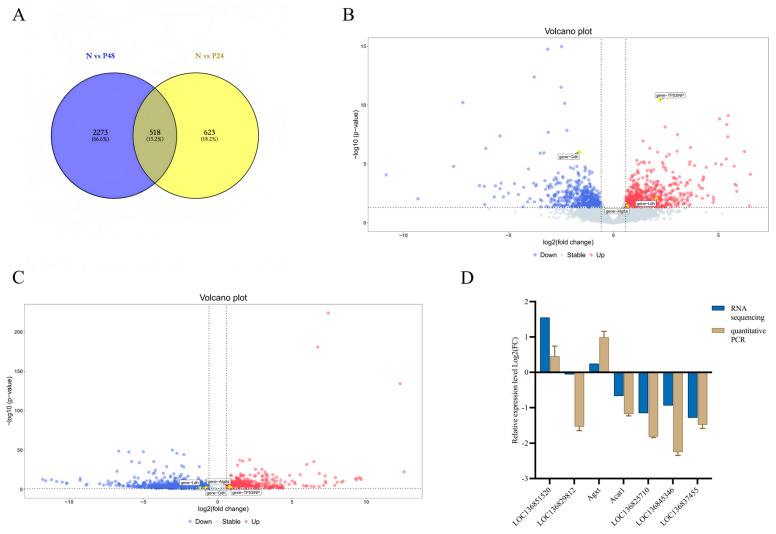
Identification of DEGs under high-salinity stress and RT-qPCR validation. (**A**): Venn diagram illustrating the number of shared and unique DEGs between different comparison groups. (**B**): Volcano plot showing the distribution of DEGs in the N vs. P24 comparison. (**C**): Volcano plot showing the distribution of DEGs in the N vs. P48 comparison. (**D**): Correlation between RNA-seq and RT-qPCR results. Comparison of the expression fold-changes of selected mRNAs derived from sequencing and RT-qPCR.

RNA-Seq analysis of the hepatopancreas from the control (N) and high-salinity stressed (P24, P48) *M. rosenbergii* generated 57.82 Gb of high-quality clean data (Q30 > 93.35%), yielding 25,154 assembled unigenes (Supplementary Table S2). As illustrated by the Venn diagram, a distinct duration-dependent increase in DEG abundance was observed (Figure 1A). Specifically, 1141 DEGs (611 upregulated, 530 downregulated) were identified in the N vs. P24 comparison (Figure 1B), which increased to 2791 DEGs (1041 upregulated, 1750 downregulated) by 48 h (Figure 1C). Notably, genes regulating homeostasis (*Atg8a*, *TP53INP*) and core metabolic enzymes (*Gdh*, *Ldh*) exhibited distinct time-course expression patterns (Supplementary Table S3). Furthermore, RT-qPCR validation of seven randomly selected DEGs demonstrated expression trends highly consistent with the RNA-Seq results (Figure 1D), confirming the reliability of the transcriptomic dataset for downstream analyses.

### 3.2. KEGG Enrichment and Temporal Shifts in the Regulatory Networks of DEGs

**Figure 2:**
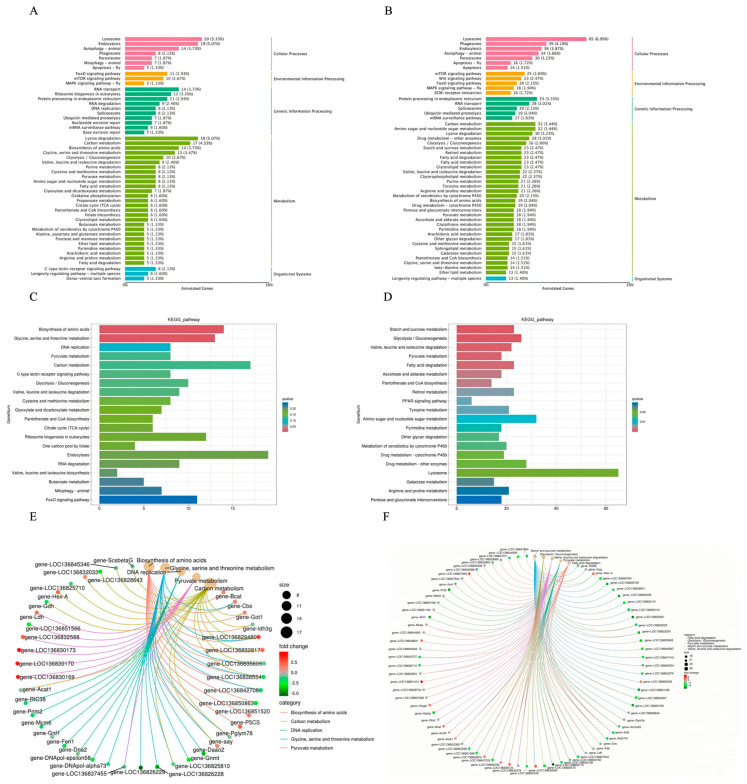
KEGG pathway enrichment analysis and regulatory network construction of DEGs under high-salinity stress. (**A**): KEGG classification of DEGs in the N vs. P24 group. (**B**): KEGG classification of DEGs in the N vs. P48 group. (**C**): KEGG enrichment bar plot of DEGs in the N vs. P24 group. (**D**): KEGG enrichment bar plot of DEGs in the N vs. P48 group. (**E**): KEGG enrichment network of DEGs in the N vs. P24 group. (**F**): KEGG enrichment network of DEGs in the N vs. P48 group.

KEGG enrichment analysis was performed to evaluate temporal shifts in metabolic pathways. At 24 h of stress, DEGs were significantly enriched (*p* < 0.05) in the “biosynthesis of amino acids” and immune-related pathways (e.g., autophagy, lysosome, and the FoxO signalling pathway). In the core regulatory network, *Got1*, *Gdh*, and *Ldh* were identified as hub genes, whereas DNA replication processes were distinctly suppressed (Figure 2A,C,E). By 48 h, the enrichment focus shifted toward “ascorbate and aldarate metabolism” and “retinol metabolism” (Figure 2B,D). The network hubs transitioned to *Aldh*, *Aldh7A1*, and *Acat1*, reflecting an enhanced degradation and utilization of branched-chain amino acids and fatty acids (Figure 2F, Supplementary Table S3). Notably, initially upregulated genes, such as *Ldh* and *Eno*, were subsequently downregulated at 48 h.

### 3.3. Untargeted Metabolomic Analysis

#### 3.3.1. Metabolomic Profiling and Identification of DMs Under High-Salinity Stress

**Figure 3:**
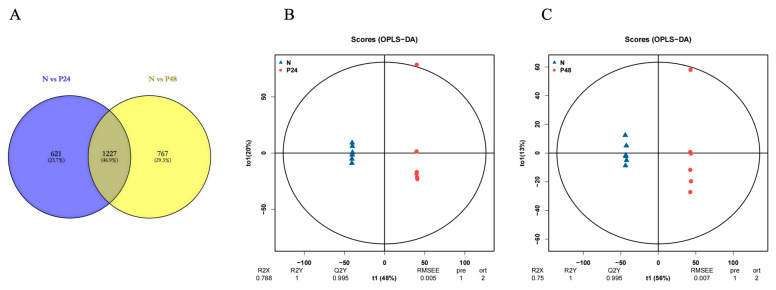
Metabolomic analysis of the hepatopancreas in *M. rosenbergii* under salinity stress. (**A**): Venn diagram illustrating the number of shared and unique DMs between different comparison groups. (**B**): OPLS-DA score plot for the N vs. P24 group. (**C**): OPLS-DA score plot for the N vs. P48 group.

To ensure the reliability of our metabolomic findings, we first assessed the quality of the OPLS-DA models for all comparison groups. The models exhibited strong explanatory and predictive power: for N vs. P24, R^2^X = 0.788, R^2^Y = 1, and Q^2^ = 0.995 (Figure 3B); for N vs. P48, R^2^X = 0.75, R^2^Y = 1, and Q^2^ = 0.995 (Figure 3C). Permutation tests (200 iterations) confirmed the robustness of these models and the absence of overfitting (*p* < 0.05 in all cases). Following validation, metabolomic analysis of the hepatopancreas from control (N) and high-salinity stressed (P24, P48) *M. rosenbergii* revealed a duration-dependent increase in the number of differentially expressed metabolites (DMs) (Figure 3A). Compared to the control, 1848 DMs (922 upregulated, 926 downregulated) were identified at 24 h (N vs. P24), and this number increased to 1994 DMs (1266 upregulated, 728 downregulated) at 48 h (N vs. P48).

#### 3.3.2. KEGG Enrichment and Temporal Shifts in the Regulatory Networks of DMs

**Figure 4:**
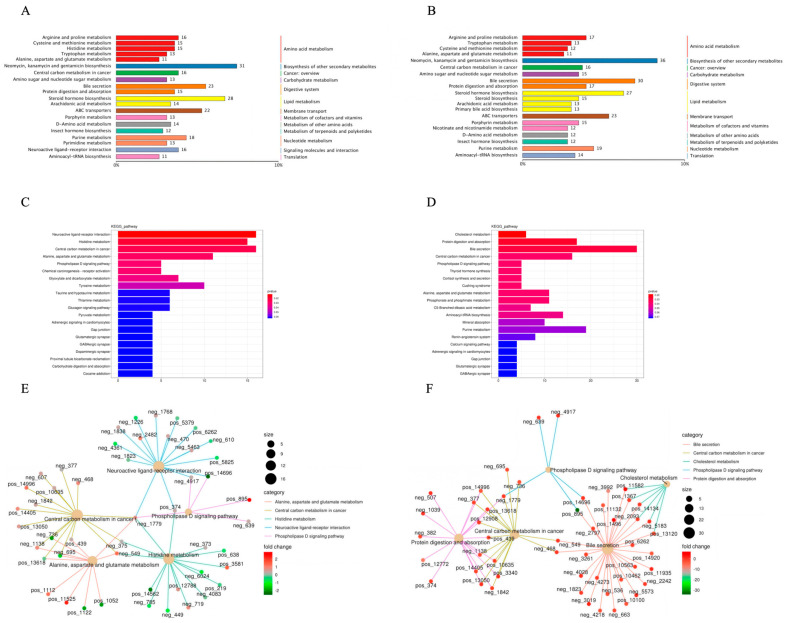
(**A**): KEGG classification of DMs in the N vs. P24 group. (**B**): KEGG classification of DMs in the N vs. P48 group. (**C**): KEGG enrichment bar plot of DMs in the N vs. P24 group. (**D**): KEGG enrichment bar plot of DMs in the N vs. P48 group. (**E**): KEGG enrichment network of DMs in the N vs. P24 group. (**F**): KEGG enrichment network of DMs in the N vs. P48 group.

KEGG enrichment analysis of DMs revealed a dynamic transition in metabolic mobilization across the stress duration. At 24 h of stress, DMs were broadly mapped to nucleotide and amino acid metabolism pathways (Figure 4A). Notably, eight pathways were significantly enriched (*p* < 0.05), including the phospholipase D signalling pathway and alanine, aspartate, and glutamate metabolism (Figure 4C). The correlation network demonstrated that the upregulated hub metabolite L-glutamate (neg_1779) interconnected five core processes, encompassing the phospholipase D signalling pathway, amino acid metabolism, and central carbon metabolism. Simultaneously, central carbon and amino acid metabolism were closely interconnected via cross-pathway molecules such as L-glutamine (pos_439) (Figure 4E).

By 48 h, the enrichment focus shifted toward purine metabolism, steroid hormone biosynthesis, arginine and proline metabolism, and central carbon metabolism (Figure 4B). Statistical analysis showed that 12 pathways were significantly enriched (*p* < 0.05) at this stage. These included previously retained pathways (e.g., the phospholipase D signalling pathway and alanine, aspartate, and glutamate metabolism), alongside newly enriched processes like cortisol synthetase and secretion, and cholesterol metabolism (Figure 4D). Within the 48 h regulatory network, L-glutamate (neg_1779) remained a core hub molecule, effectively bridging the phospholipase D signalling pathway, central carbon metabolism, and protein digestion and absorption. Notably, unlike the balanced regulation seen initially, the vast majority of network metabolites were predominantly upregulated at this stage, with the exception of specific molecules such as 3′,5′-Cyclic AMP (pos_895) (Figure 4F).

### 3.4. Results of Integrated Transcriptomic and Metabolomic Analysis

#### 3.4.1. KEGG Co-Enrichment Analysis of DEGs and DMs

**Figure 5:**
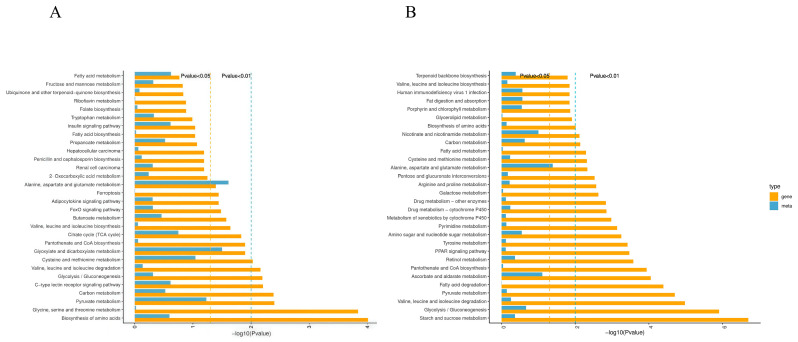
Integrated transcriptomic and metabolomic analysis of the hepatopancreas in *M. rosenbergii* under salinity stress. (**A**): KEGG enrichment bar plot of the top 30 significantly enriched pathways for DEGs and DMs in the N vs. P24 comparison. (**B**): KEGG enrichment bar plot of the top 30 significantly enriched pathways for DEGs and DMs in the N vs. P48 group.

To investigate the core regulatory mechanisms of the salinity stress response, DEGs and DMs from the 24 h and 48 h time points were subjected to KEGG co-enrichment analysis. During the initial stress phase (24 h), transcriptional and metabolic responses primarily converged on energy-balancing processes, including pyruvate metabolism, carbon metabolism, and glycolysis/gluconeogenesis (Figure 5A). By 48 h, the co-enriched pathways expanded to encompass fatty acid degradation, retinol metabolism, the PPAR signalling pathway, and starch and sucrose metabolism (Figure 5B). This metabolic expansion reflects an enhanced mobilization of broader lipid and carbohydrate energy reserves under prolonged high-salinity conditions. Notably, “alanine, aspartate, and glutamate metabolism” remained significantly enriched (*p* < 0.05) at both time points, highlighting its critical role as a central metabolic hub throughout the stress response.

#### 3.4.2. In-Depth Characterisation of the Key Hub Pathway: “Alanine, Aspartate, and Glutamate Metabolism”

**Figure 6:**
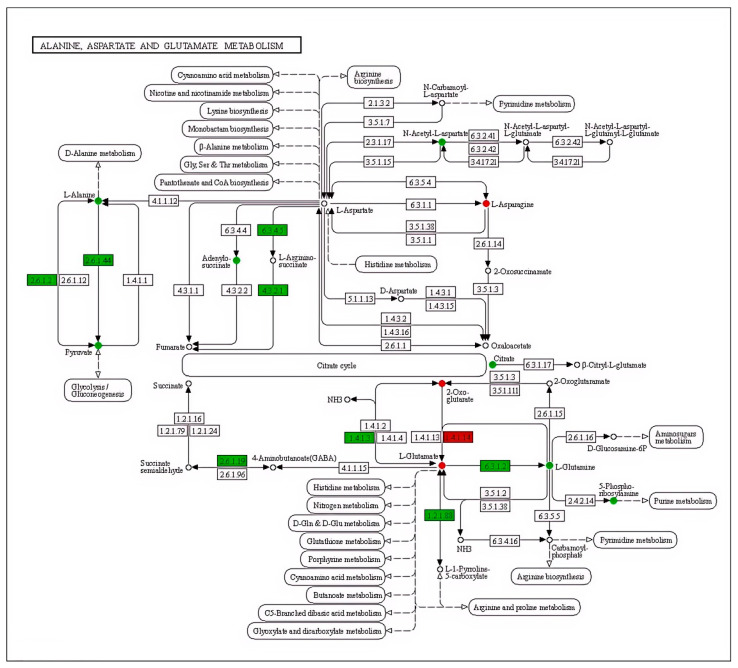
Mapping of DEGs and DMs involved in “alanine, aspartate, and glutamate metabolism” onto the KEGG pathway map. Boxes indicate gene products, and circles indicate metabolites. Colored backgrounds mark the significant DEGs and DMs: red for up-regulation, green for down-regulation, and blue for nodes containing both up- and down-regulated entities.

To further analyse this central metabolic hub, an interaction network comprising 11 genes and 11 metabolites was reconstructed (Figure 6, Supplementary Table S4). Under prolonged high-salinity stress (48 h), this pathway exhibited distinct transcriptional repression and metabolic flux redirection. Transcriptionally, the majority of key rate-limiting enzymes were significantly downregulated. Specifically, the expression of alanine-glyoxylate aminotransferase (*Agxt*), urea cycle-related argininosuccinate synthase/lyase (*Ass*, *Argl*), and core metabolic enzymes glutamine synthetase (*Gs*) and glutamate dehydrogenase (*Gdh*) was substantially suppressed. Metabolically, core metabolites including L-glutamate, oxoglutaric acid, and L-asparagine significantly accumulated. Conversely, L-alanine and L-glutamine (acting as energy substrates and osmo-effectors), alongside tricarboxylic acid (TCA) cycle intermediates (e.g., pyruvic acid and citric acid), were markedly depleted. Ultimately, these findings confirm that “alanine, aspartate, and glutamate metabolism” constitutes a critical regulatory nexus for *M. rosenbergii* during acute salinity stress, coordinating immune defence, energy balance, and osmotic homeostasis by modulating carbon and nitrogen fluxes.

## 4. Discussion

Although adult *M. rosenbergii* exhibit considerable euryhalinity (0~25 ppt) [32], acute salinity spikes (e.g., 20‰) can exceed their osmoregulatory capacity, triggering severe physiological stress. Previous studies indicate that phenotypic impairments induced by hypersalinity, such as growth retardation and delayed reproduction, fundamentally stem from cellular-level metabolic perturbations and immune imbalances [6]. To uncover these underlying mechanisms, the present study employed an integrated transcriptomic and metabolomic approach to systematically delineate the dynamic molecular responses of *M. rosenbergii* to acute high-salinity stress. Our multi-omics data demonstrate the time-dependent cumulative effects of salinity stress: the abundance of DEGs (2791) and DMs (1994) at 48 h substantially exceeded those at 24 h (1141 DEGs and 1848 DMs). Crucially, this massive expansion of the responsive molecular network is not merely a quantitative accumulation but carries profound biological significance for osmoregulatory adaptation. It signifies a strategic transition from an initial acute metabolic mobilization (24 h) to a subsequent systemic homeostatic remodelling (48 h), aligning with the findings of Wang [33]. Specifically, this coordinated transcriptomic and metabolomic recalibration allows the organism to prioritize the retention of critical osmo-effectors while suppressing energy-exhaustive pathways. Ultimately, this study highlights a potential transcriptional-metabolic decoupling phenomenon, alongside coordinated cytoprotective responses, as key responses to counteract continuous osmotic fluctuations, thereby providing a theoretical framework for understanding crustacean stress resilience and facilitating the selective breeding of salt-tolerant strains.

### 4.1. Autophagic and p53-Mediated Cytoprotection

Within this mobilized molecular network, maintaining intracellular quality control is essential for organismal survival. Our data indicate that by 48 h, *M. rosenbergii* exhibits significant changes in autophagy-related gene expression alongside the activation of the p53 signalling pathway to mount a cellular defence. Beyond its fundamental role in crustacean innate immunity [34,35,36], autophagy functions as a critical mechanism for intracellular clearance and metabolic recycling under environmental duress. Notably, suppressing *Atg*-related genes in Danio rerio heightens susceptibility to bacterial infection [37]. Similarly, *Atg* transcripts have been identified as immune modulators during hypoxia and pathogen exposure in *Macrobrachium nipponense* [38] and *Eriocheir sinensis* [39]. Consistent with observations by Sui [40], we found that *Atg8a* [41]—a core driver of autophagosome formation—was upregulated at 48 h. However, our comprehensive transcriptomic profiling revealed a more complex scenario: the autophagy initiation factor *Atg1* was downregulated, and other core autophagy markers (such as *Atg5*, *Atg12*, and *Beclin-1*) remained stably expressed without significant differential regulation. Concurrently, downstream genes involved in vesicle trafficking and lysosomal degradation (e.g., *cathD*, *Rab*, and *Vps* families) were actively differentially expressed. Therefore, rather than a full-scale classic autophagic activation, this specific transcriptional mobilization during prolonged hypersalinity likely facilitates the clearance of osmotically induced misfolded proteins and oxidized macromolecules, while degrading cellular components to replenish emergency metabolic substrate reserves.

Concurrently, the p53 signalling pathway is central to maintaining genomic stability and facilitating DNA repair [42]. In species such as *Anabas testudineus* [43] and *Trachemys scripta elegans* [44], *p53* expression is induced during salinity stress, mitigating cellular damage by modulating apoptosis. Furthermore, this pathway is involved in immune defence; for instance, it mediates viral resistance in *Micropterus salmoides* [45] and is upregulated in *Takifugu obscurus* following *Vibrio parahaemolyticus* infection [46]. These observations highlight the p53 pathway as a regulatory hub integrating environmental stress tolerance and immune defence in aquatic organisms. In the present study, the core *p53* target gene *TP53INP* was upregulated at 48 h, indicating the activation of DNA repair programs or cell cycle arrest in response to hypersalinity-induced DNA damage [47]. The concurrent activation of autophagy and the p53 pathway demonstrates that *M. rosenbergii* mounts a comprehensive cytoprotective response, integrating misfolded protein clearance with genomic repair under severe osmotic stress.

### 4.2. Transcriptional-Metabolic Discordance and Potential Metabolic Imbalance

Beyond serving as fundamental substrates for protein synthesis, amino acids function as effector molecules for maintaining cellular homeostasis [48]. Our integrated multi-omics analysis revealed that “alanine, aspartate, and glutamate metabolism” was significantly enriched under salinity stress, highlighting its role as a core regulatory hub in the environmental adaptation of *M. rosenbergii*. Within this pathway, *Gdh*—a key node linking amino acid and carbohydrate metabolism—exhibited “transcriptional-metabolic decoupling”. While *Gdh* is typically upregulated to promote amino acid synthetase during salinity stress in crustaceans such as *Eriocheir sinensis* [49], our study found that *Gdh* transcription was suppressed at 48 h, juxtaposed with an accumulation of L-glutamate. This apparent divergence between gene transcription and metabolite abundance suggests a potential transcriptional-metabolic discordance [10]. We hypothesize that by suppressing *Gdh* expression, *M. rosenbergii* might restrict the oxidative degradation of glutamate, thereby potentially preserving the intracellular pool of this crucial osmo-effector. Furthermore, sustaining an elevated glutamate pool is essential for synthesizing the antioxidant glutathione (GSH) to combat oxidative stress [50]. Considering that 20‰ approaches the physiological tolerance threshold of *M. rosenbergii*, the global transcriptional repression induced by prolonged severe stress may also contribute to the observed downregulation of *Gdh*.

This discordance may reflect a physiological trade-off between metabolite accumulation and energy expenditure. The intracellular accumulation of free amino acids (FAAs) constitutes a primary cellular defence against hyperosmotic environments [51]. As key organic osmolytes, alanine, aspartate, and glutamate are crucial for maintaining intra- and extracellular osmotic balance and cell volume stability [52]. However, because osmoregulation is an energy-intensive process, the activation of alternative energy-yielding pathways is necessitated. Previous studies highlight the importance of amino acid catabolism in aquatic animals; for example, the oxidative metabolism of glutamate, glutamine, and aspartate can contribute 60–70% of total ATP production in the tissues of teleosts [53]. In the present study, the inferred restriction of the primary *Gdh*-mediated glutamate catabolic pathway, concurrent with the downregulation of the key glycolytic gene *Ldh*, likely necessitated compensatory energy production from alternative amino acids. Consequently, alanine, a major gluconeogenic substrate, was utilized to sustain energy reserves [54]. Concurrently, aspartate and other amino acids were converted into α-ketoglutarate or oxaloacetate via transamination or deamination, feeding directly into the tricarboxylic acid (TCA) cycle through anaplerotic reactions to fuel ATP synthesis [55,56]. Ultimately, this carbon-nitrogen metabolic shunting accommodates local osmotic homeostasis while safeguarding the baseline energy turnover of *M. rosenbergii* during prolonged high-salinity stress.

Beyond coordinating osmoregulation and energy homeostasis, this pathway mediates metabolism-immunity cross-talk during the innate immune response [57]. Sustaining an elevated glutamate pool serves dual physiological functions: it acts as a primary osmo-effector and provides the essential precursor for glutathione (GSH) biosynthesis, which is vital for mitigating hypersalinity-induced oxidative stress. Previous studies demonstrate that exogenous glutamate supplementation enhances intestinal antioxidant capacity and disease resistance in aquatic animals [58]. Similarly, aspartate improves survival during pathogenic challenge by driving metabolic reprogramming [59,60]. Consequently, the sustained enrichment of “alanine, aspartate, and glutamate metabolism” underscores the multifaceted utility of these amino acids in *M. rosenbergii*, functioning concurrently as physical osmolytes and biochemical immune substrates. These observations align with findings in *Exopalaemon carinicauda* under salinity stress [61]. Ultimately, this conserved immunomodulatory mechanism fortifies crustacean resilience against extreme environmental fluctuations.

In summary, our data suggest a potential metabolic conservation strategy under hyperosmotic stress. In this hypothetical scenario, the downregulation of *Gdh* may act as a metabolic bottleneck, restricting amino acid catabolism and thereby helping to conserve glutamate as a crucial osmo-effector. Simultaneously, the inferred redirection of carbon skeletons from amino acids into the TCA cycle could provide an essential energy boost for osmotic regulation. Furthermore, the elevated glutamate pool might facilitate GSH synthesis, serving as a secondary defense mechanism to mitigate salinity-induced oxidative stress. This integrated metabolic network, characterized by potential transcriptional-metabolic decoupling, likely represents a key adaptive mechanism by which *M. rosenbergii* maintains cellular integrity and resilience against extreme salinity.

### 4.3. Lipid and Nucleotide Reprogramming for Metabolic Compensation

Beyond the aforementioned amino acid metabolic imbalance, the reprogramming of lipid and nucleotide metabolism provides metabolic compensation for *M. rosenbergii* during high-salinity stress. Metabolomic profiling revealed an enrichment of the phospholipase D (PLD) signalling pathway, whose lipid products drive protein and carbohydrate metabolism via mTOR pathway activation [62,63]. Concurrently, cholesterol is mobilized to synthesize ecdysteroid hormones [64,65,66,67], orchestrating cell membrane remodelling and osmotic adaptation through endocrine signalling. Furthermore, the activation of purine and pyrimidine metabolism [68] supplies precursors for the aforementioned p53-mediated DNA repair, while replenishing the cellular ATP pool. This energetic replenishment directly fuels highly demanding transmembrane ion transport processes (e.g., Na^+^/K^+^-ATPase) under hyperosmotic conditions. Taken together, lipid and nucleotide reprogramming is closely linked with the amino acid osmoregulatory network, establishing a stress resilience mechanism in *M. rosenbergii* that integrates energy compensation, structural repair, and physiological homeostasis. Importantly, the energetic demand for osmotic homeostasis is largely driven by transmembrane ion transporters, most notably the Na^+^/K^+^-ATPase, which consumes a significant portion of cellular ATP. Our findings suggest a synergistic mechanism: the metabolic reconfiguration observed here—characterized by the redirection of amino acid-derived carbon skeletons into the TCA cycle—provides the necessary ATP pool to sustain high-intensity Na^+^/K^+^-ATPase activity. This integration allows *M. rosenbergii* to maintain transmembrane ion gradients and cell volume stability despite the severe osmotic pressure.

### 4.4. Limitations of the Multi-Omics Inference

It is important to note that the transcriptional-metabolic discordance observed here—specifically the downregulation of *Gdh* alongside L-glutamate accumulation—provides correlational evidence rather than direct proof of metabolic flux. The accumulation of glutamate under high-salinity stress might also be driven by alternative mechanisms, such as increased de novo synthesis via transaminases or the GS/GOGAT pathway, reduced catabolism mediated by enzymes not fully captured in our current transcriptomic dataset (e.g., glutamate decarboxylase, GAD), or inter-organ redistribution of metabolites from other osmoregulatory tissues like gills or muscle. Consequently, this metabolic phenomenon should be rigorously regarded as a foundational hypothesis, warranting future targeted physiological investigations (e.g., enzyme activity assays or isotopic tracing) to definitively elucidate the causal networks governing amino acid flux during salinity acclimation.

## 5. Conclusions

In this study, an integrated transcriptomic and metabolomic approach was employed to characterise the molecular response of adult *M. rosenbergii* to acute high-salinity stress. Our findings indicate that prolonged stress significantly increased the abundance of DEGs and DMs in the hepatopancreas, marking a transition from an initial acute stress reaction to systemic homeostatic remodelling. This late-stage adaptation is driven by autophagy, p53 signalling, and the reprogramming of lipid and nucleotide metabolism. Furthermore, integrated analysis identified “alanine, aspartate, and glutamate metabolism” as the central metabolic hub for coping with salinity fluctuations. By modulating carbon and nitrogen fluxes, this pathway coordinates energy compensation, osmotic balance, and immune defence. Notably, the apparent transcriptional-metabolic discordance observed for key enzymes, such as *Gdh*, highlights a potential physiological phenomenon wherein the organism appears to prioritize the intracellular accumulation of osmo-effectors. Ultimately, these multi-omics insights deepen the understanding of environmental adaptation in freshwater crustaceans, providing actionable targets to enhance the stress resilience of *M. rosenbergii.* Specifically, key regulatory nodes such as *Gdh*, *Atg8a*, and *TP53INP* could potentially serve as candidate molecular markers for the selective breeding of salt-tolerant strains. Furthermore, given the central role of amino acid metabolic decoupling in osmotic adaptation, precision nutritional interventions—such as dietary supplementation with L-glutamate or L-alanine—may represent a promising strategy to bolster intracellular osmo-effector pools, mitigate oxidative damage, and ultimately improve survival rates during salinity fluctuations in regional aquaculture.

## Data Availability

This paper presents interim analytical findings based on the dataset. The complete dataset will remain available for subsequent in-depth research. Any researcher wishing to access the data to verify the results reported herein may contact the author via email xiayan1207@126.com.

## Supplementary Materials

The following supporting information can be downloaded at: https://www.mdpi.com/article/10.3390/biology15151234/s1, Table S1: Primer sequences used for RT-qPCR analysis. Table S2: Sequencing data statistics. Table S3: Summary of unique and shared differentially expressed genes (DEGs) and KEGG pathway enrichment analysis in *M. rosenbergii* at 24 h and 48 h under acute high-salinity stress. Table S4: DEGs and DMs involved in the alanine, aspartate, and glutamate metabolism pathway (ko00250).
